# High Variability in Erythrocyte, Plasma and Whole Blood EPA and DHA Levels in Response to Supplementation

**DOI:** 10.3390/nu12041017

**Published:** 2020-04-08

**Authors:** Cassandra Sparkes, Andrew J. Sinclair, Robert A. Gibson, Paul L. Else, Barbara J. Meyer

**Affiliations:** 1 School of Medicine, Molecular Horizons, University of Wollongong, Wollongong, NSW 2522, Australia; sparkesc@gmail.com (C.S.); pelse@uow.edu.au (P.L.E.); 2 Illawarra Medical Research Institute, Wollongong, NSW 2522, Australia; 3 Faculty of Health, Deakin University, Geelong, VIC 3220, Australia; Andrew.sinclair@deakin.edu.au; 4 Department of Nutrition, Dietetics and Food, Monash University, Notting Hill, VIC 3168, Australia; 5 University of Adelaide, Adelaide, SA 5005, Australia; robert.gibson@adelaide.edu.au

**Keywords:** omega-3, variability, erythrocyte, plasma, whole blood

## Abstract

(1) Aim: the aim of this secondary analysis was to report the variability in response to *n*-3 long chain polyunsaturated fatty acids (LCPUFA) supplementation in erythrocytes, plasma and whole blood of a previously published dose response study. (2) Methods: a randomized, double-blind, placebo-controlled trial of parallel design was conducted, whereby pre-menopausal women were randomly assigned to consume 0, 0.35, 0.7 or 1 g/day of supplemental eicosapentaenoic acid (EPA) plus docosahexaenoic acid (DHA). Fasted blood samples were taken at baseline and after eight weeks intervention. Erythrocyte, plasma and whole blood fatty acids were extracted using the method of Lepage and Roy and analysed using gas chromatography. (3) Results: There were significant increases in EPA plus DHA levels in the 0.7 g and 1 g dose groups, with the highest increase with the 1 g dose notably: in erythrocytes (from 5.69% to 7.59%), plasma (from 2.94% to 5.48%) and in whole blood (from 3.81% to 6.03%). There was high variability in response to the supplement in erythrocytes, plasma and whole blood across the different doses. (4) Conclusion: there is high individual variability in *n*-3 LCPUFA levels in response to *n*-3 LCPUFA supplementation, which should be taken into account in clinical trials using *n*-3 LCPUFA supplements.

## 1. Introduction

The omega-3 long chain polyunsaturated fatty acids (*n*-3 LCPUFA) are comprised of eicosapentaenoic acid (EPA), docosapentaenoic acid (DPA) and docosahexaenoic acid (DHA). DHA and arachidonic acid are important fatty acids for neurological development [1,2]. DHA is the most abundant *n*-3 LCPUFA in the brain and its role in the brain has been described in a recent review [3].

There are numerous health benefits associated with *n*-3 LCPUFA, including cardiovascular disease [4,5,6], inflammatory disease [7,8] and mental health [9,10,11,12]. Harris and von Schacky postulated that the Omega-3 Index (sum of EPA and DHA expressed as a percentage of total erythrocyte fatty acids) could be a biomarker for cardiovascular disease risk and that an omega-3 index of greater than 8% is optimal [13]. There is evidence that this omega-3 index biomarker may also be useful in terms of mental health [9,10,11,14].

There are many factors that are associated with *n*-3 LCPUFA levels [15]. Factors positively associated with the *n*-3 LCPUFA levels are age and sex (women less than 50 years of age). Factors negatively associated with the *n*-3 LCPUFA levels are: genetics, BMI (if erythrocyte EPA and DHA is less than 6%), smoking and alcohol. Therefore, the International Society for the Study of Fatty Acids and Lipids (ISSFAL) has released a position statement, which states that any type of *n*-3 LCPUFA research should measure *n*-3 LCPUFA levels in the blood [16].

There have been numerous trials investigating the effect of *n*-3 LCPUFA on various outcome measures, but only a few have reported the large individual variability in response to supplementation [17,18,19,20].

Therefore, the aim of this study was to report the variability in response to *n*-3 LCPUFA supplementation, as a secondary analysis of a dose response study.

## 2. Materials and Methods

### 2.1. Study Participants

Participants in this study were a subset of 53 premenopausal women reported elsewhere with respect to the effect of DHA supplementation on plasma triglycerides and lipoproteins [21]. This cohort of 45 participants (85% of total sample) represents those for whom data on erythrocyte, plasma and whole blood fatty acid compositions were available for secondary analysis.

Healthy premenopausal women with mildly elevated triglycerides (>1.0 mmol/L) and regular menstrual cycles (28–32 days) were recruited for this study, conducted at the University of Wollongong, Australia. Exclusion criteria included age <18 or >40 years, consumption of fish oil supplements, and known existing cardiovascular disease. Subjects who completed the trial were required to achieve a capsule compliance rate of >90% on the basis of self-report and excess capsule count, and a body weight change of ≤5% from baseline. The study protocol was approved by the Human Research Ethics Committee of the University of Wollongong (HE06/317), and the research has therefore been performed in accordance with the ethical standards laid down in the 1964 Declaration of Helsinki and its later amendments. Written informed consent was obtained from the participant prior to inclusion in the study (Australian and New Zealand Clinical Trial Registration ID: ANZCTRN12607000566437).

### 2.2. Study Design

A randomized, double-blind, placebo-controlled trial of parallel design was conducted, whereby subjects were randomly assigned to consume 0, 350, 700, or 1000 mg/day of supplemental EPA plus DHA for two menstrual cycles (approximately 8 weeks). Randomization was controlled for age, BMI, and contraceptive pill use.

DHA was provided in the form of HiDHA™ tuna oil (500 mg sized capsules supplied by Nu-Mega Ingredients, Australia). Subjects consumed six capsules daily, with the four different doses achieved by varying the proportion of active (HiDHA™ tuna oil) and placebo (Sunola oil, 500 mg) capsules for each dose group. Each tuna oil capsule provided 35 mg EPA and 135 mg DHA, whereas each placebo capsule provided 355 mg monounsaturated fatty acids (MUFA), 55 mg saturated fatty acids (SFA), and 14 mg PUFA (no *n*-3 LCPUFA).

Daily doses of capsules were provided in individual zip-lock bags, and an excess of these bags was distributed to participants. Participants also kept a diary to record daily capsule intake and menstrual cycle status. Compliance with capsule consumption was assessed using the diary records and excess capsule count, and confirmed by measurement of erythrocyte *n*-3 LCPUFA levels.

### 2.3. Clinic Visits

Study participants monitored their menstrual cycle and reported commencement of menses, such that they attended the research clinic on two consecutive mornings between days 3–5 of their menstrual cycle, both at baseline and after approximately 8 weeks (two menstrual cycles) of DHA supplementation. Height (baseline only), body weight, blood pressure and fasted blood samples were collected at baseline and at the end of the intervention. Blood pressure was measured in triplicate at all clinic visits, using an automatic blood pressure monitor.

### 2.4. Dietary Intake Analysis

Comprehensive information on the habitual intakes of PUFA, including individual *n*-3 LCPUFA, was obtained using a validated electronic PUFA food frequency questionnaire [22]. The Victorian Anti-Cancer Council Food Frequency Questionnaire was administered to determine the macro- and micro-nutrient intake levels of participant’s whole diets. Participants were instructed to avoid dietary changes for the study duration.

### 2.5. Blood Sample Collection and Processing

Following an overnight fast (>10 h), venous blood was collected into ethylenediaminetetraacetic acid (EDTA) tubes on two consecutive days (36 mL on Day 1; 9 mL on Day 2) at baseline and post-intervention. An aliquot of whole blood was stored at −80 °C and the remaining blood samples were subjected to centrifugation 2000g at 4 °C for 10 minutes, to separate plasma from erythrocytes. Aliquots of erythrocytes, plasma and whole blood were stored at −80 °C for the analysis of fatty acids; fatty acids were recorded once both at baseline and post-intervention.

### 2.6. Fatty Acid Analysis

Fatty acid analyses of erythrocytes, plasma and whole blood were performed on total lipids, without separation of the different lipid classes. The fatty acid profiles of erythrocytes were determined using standard methods previously reported [23]. Briefly, erythrocyte aliquots (400 μL) were thawed and re-suspended in a TRIS buffer (10 mM Bis Tris, 2 mM EDTA Na2, pH 7.2), at room temperature for 30 min. The samples were then spun in an ultracentrifuge at 315,000 g for 30 min at 4 °C (Beckman L-80 OPTIMA, Indianapolis, IN, USA) to pellet erythrocyte membranes. Upon removal of the supernatant, the erythrocyte membrane pellet was re-suspended in 200 μL of distilled water. A fixed volume (150 μL) of the erythrocyte membrane suspension was used for the direct transesterification of fatty acids [24], using heneicosanoic acid as the internal standard, as described below.

Preparation of fatty acid methyl esters (FAME) from plasma fatty acids was performed using the one-step direct transesterification method of Lepage and Roy [24]. During this process, only the complex lipids will be transmethylated, and the free fatty acids will be methylated. Briefly, 200 μL plasma was added to Teflon-lined 10 mL glass screw-cap tubes, followed by the addition of 2 mL of methanol/toluene (4:1 v/v) containing BHT as an antioxidant (0.01% w/v), and 200 μL of 0.2 mg/mL internal standard solution (heneicosaenoic acid, 21:0), Sigma Aldrich, Castle Hill, Australia). Acetyl chloride (200 μL) as a catalyst was then added slowly using a positive displacement pipette, while vortexing tubes. Sealed tubes were then heated to 100 °C for 1 h on a heat block (Thermoline BTC-9090 Dry Block Heater, Thermoline Scientific Equipment, Smithfield, Australia). Tubes were then cooled, followed by a slow addition of 5 mL of 6% potassium carbonate while vortexing. Tubes were then subjected to centrifugation for 10 min at 2000 g and 4 °C (Rotina 46R, Hettich Zentrifugen, Rotanta 460 Centrifuge, GMI In., Minnesota, MN, USA). The supernatant (toluene phase) was then collected and stored in a small glass autosampler vial (with insert), for gas chromatography analysis. Whole blood fatty acids were extracted as outlined above for plasma fatty acids.

FAME were analysed by injecting 1 μL of each sample into a gas chromatograph (GC 17A Shimadzu, Columbia, MD, USA) equipped with an autoinjector, 30 m FAME capillary column (0.25 mm internal diameter, Varian, Palo Alto, CA, USA), and flame ionization detector. Hydrogen was used as the carrier gas. Fatty acid peaks were identified by comparison to known mixed standards (Nu-Chek Prep, Minnesota, USA; Sigma Aldrich, Castle Hill, Australia), and quantified using Shimadzu software (Class-VP 7.2.1 SP1, Kyoto, Japan). The data is presented as percentage of total erythrocyte fatty acids, plasma fatty acids and whole blood fatty acids.

### 2.7. Statistical Analysis

Statistical analyses were conducted using JMP 5.1 statistical software (SAS, Cary, NC, USA). The Shapiro–Wilk test was used to assess whether each variable fit a normal distribution. Non-normal variables were subsequently transformed using the log_10_ algorithm prior to statistical analyses. Baseline and post-supplementation differences between dose groups were examined using a one-way ANOVA with Tukey’s honest significant difference (HSD) analysis. Inter-individual variability in response (i.e., the coefficient of variation) was calculated per dose group as the standard deviation divided by the mean for erythrocytes, plasma and whole blood. A value of *p* < 0.05 was considered statistically significant for all analyses.

## 3. Results

### 3.1. Study Participants Characteristics

There were no significant differences between dose groups in age, body mass index (BMI), systolic or diastolic blood pressures (Table 1). Blood pressures and BMI were within the healthy range, with the latter being borderline overweight on average.

### 3.2. Dietary Intakes and Compliance to Treatment

Dietary macronutrient and fatty acid intakes of all participants combined, and for each dose group, are displayed in Table 2. Total PUFA intake in the 0.35 g/day group was significantly greater than the placebo group, explained by a greater intake of linoleic acid. It appeared this was due to an unusually high consumption of oils by one participant in that dose group, and of nuts by another. The groups did not differ in their habitual dietary intakes of any other PUFA, including EPA and DHA.

For EPA and DPA, the mean and median intakes did not differ substantially. However, for the study cohort combined, the median DHA intake was approximately one third lower than the mean intake (0.179 g/day). This was due to the skewed distribution of fish and seafood intake, whereby some participants reported regularly consuming no fish or seafood.

All participants consumed ≥90% of their allocated capsules and therefore all participants were compliant.

### 3.3. Variability of Baseline Dietary EPA Plus DHA (g/day) and Erythrocyte EPA Plus DHA (% of Total Fatty Acids)

**Figure 1:**
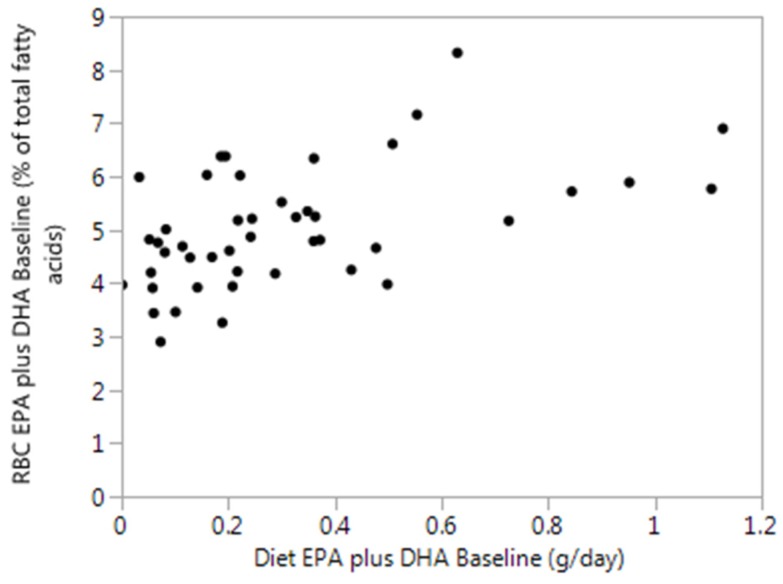
Dietary eicosapentaenoic acid (EPA) plus docosahexaenoic acid (DHA) intakes (g/day) versus erythrocyte EPA plus DHA (expressed as % of total fatty acids) from all participants (*n* = 45) at baseline.

Figure 1 shows the high variability of erythrocyte EPA plus DHA levels, prior to supplementation across a wide range of dietary intakes. The coefficient of variation of erythrocyte EPA plus DHA levels is high (0.22). The coefficient of variation of plasma was 0.32 and whole blood was 0.29.

### 3.4. Fatty Acid Response to n-3 LCPUFA Supplementation

There were no significant differences in the placebo group between baseline and post-supplementation fatty acids in erythrocytes (*p* = 0.68), plasma (*p* = 0.70) and whole blood (*p* = 0.67).

There were significant increases in erythrocyte, plasma and whole blood fatty acids in the groups supplemented with 0.7 g and 1 g/day, with the greatest increase with the 1 g dose (Table 3).

As expected, the 3 measures of fatty acid assessments (erythrocyte, plasma and whole blood) were highly correlated. The correlations of erythrocyte EPA plus DHA (expressed as percent of total fatty acids) with plasma and whole blood at baseline were 0.87 and 0.88, respectively. The correlation of plasma EPA plus DHA (expressed as percent of total fatty acids) with whole blood at baseline was 0.93. The correlations of erythrocyte EPA plus DHA (expressed as percent of total fatty acids) with plasma and whole blood post-intervention were 0.90 and 0.90, respectively. The correlation of plasma EPA plus DHA (expressed as percent of total fatty acids) with whole blood post-intervention was 0.97.

Including BMI in the model, the model improved slightly, explaining up to 55% to 58% of the variance of EPA plus DHA in the blood (erythrocytes, plasma and whole blood), whereas the BMI in addition to dietary intakes of EPA plus DHA at baseline explained between 26% to 32% (Table 4). In model 2, the correlation with BMI was negative so people with a higher BMI had lower EPA plus DHA levels.

### 3.5. Individual Response to n-3 LCPUFA Supplementation

**Figure 2:**
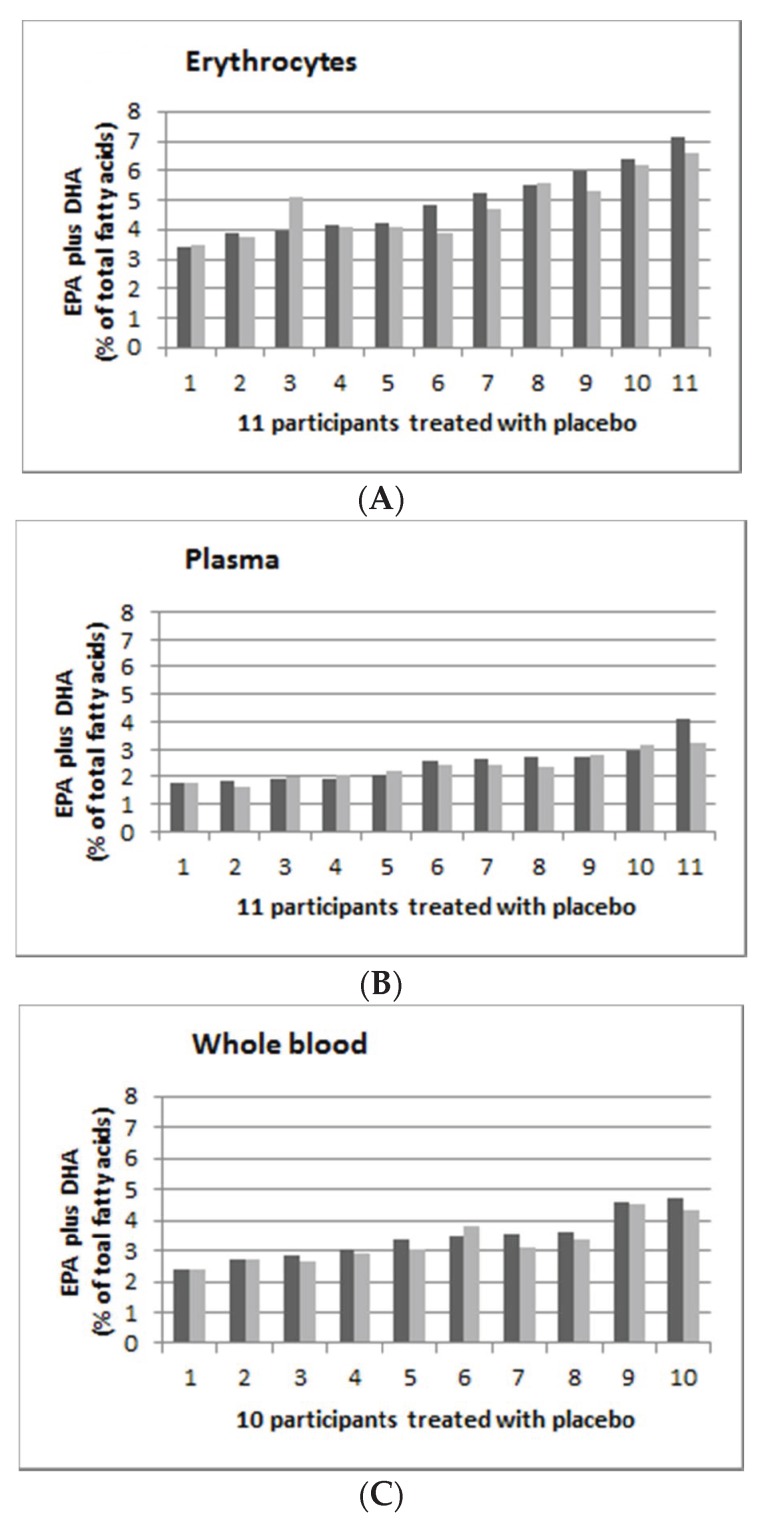
Omega-3 indices (EPA plus DHA expressed as percent of total fatty acids) in ascending order of 11 participants from the placebo group (0 mg EPA plus DHA per day). (**A**): Erythrocyte; (**B**): Plasma; and (**C**): Whole blood. Dark grey is baseline (week 0) and light grey is post-supplementation (week 8).

The individual’s response to supplementation is highly variable. Figure 2 shows the individual persons’ EPA and DHA (as % of total fatty acids), supplementation with placebo in erythrocytes (Figure 2A), plasma (Figure 2B) and whole blood (Figure 2C) with pre-supplementation (in dark grey) and post-supplementation (in light grey). The range of post-supplementation minus pre-supplementation for erythrocyte, plasma and whole blood was −0.96 to 1.13, −0.85 to 0.19 and −0.45 to 0.33 for erythrocyte, plasma and whole blood, respectively.

**Figure 3:**
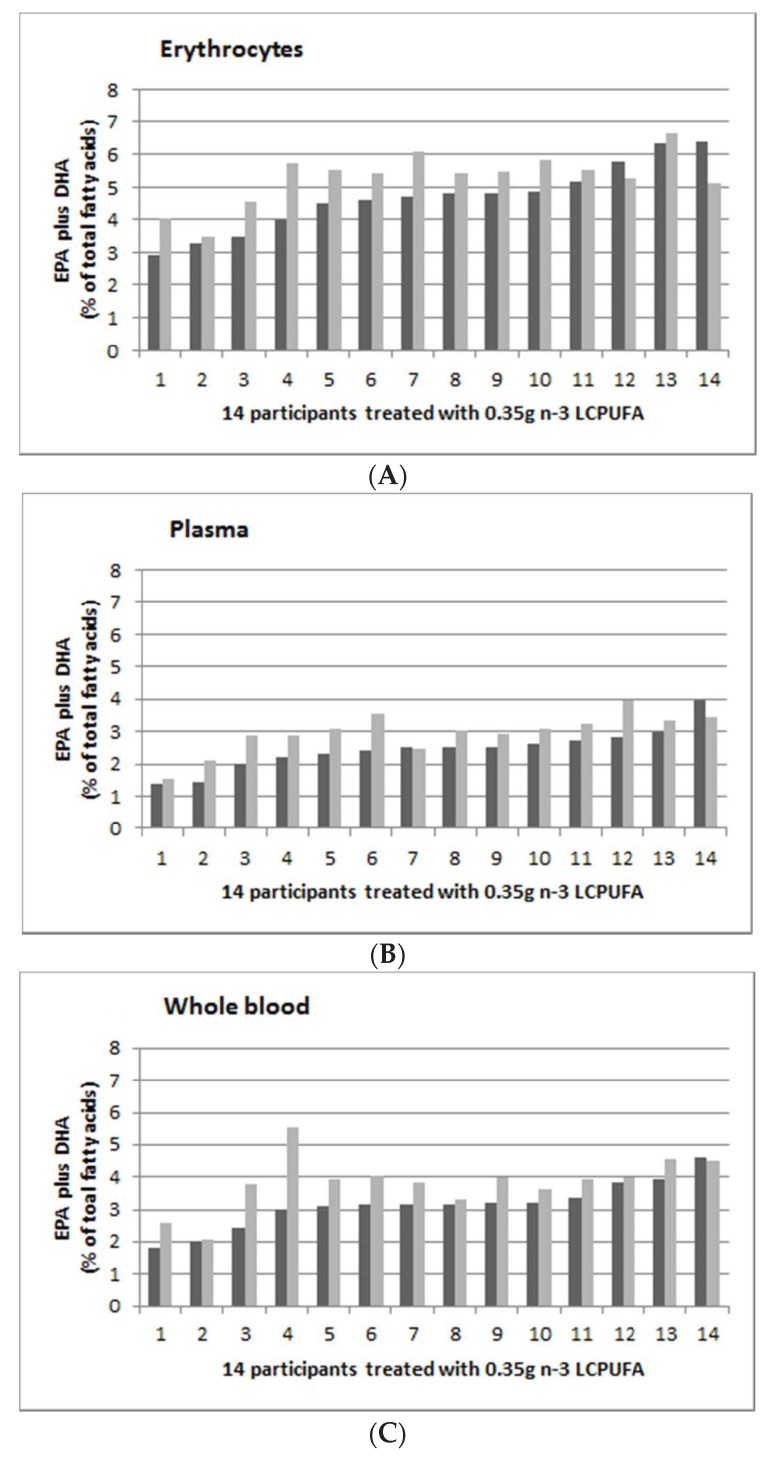
Omega-3 indices (EPA plus DHA expressed as percent of total fatty acids) in ascending order of 14 participants from the 0.35 g group (0.08 g EPA plus 0.27 g DHA per day). (**A**): Erythrocyte; (**B**): Plasma; and (**C**): Whole blood. Dark grey is baseline (week 0) and light grey is post-supplementation (week 8).

Figure 3 shows the individual person’s EPA and DHA (as % of total fatty acids) supplementation with 0.35 g *n*-3 LCPUFA per day in erythrocytes (Figure 3A), plasma (Figure 3B) and whole blood (Figure 3C), with pre-supplementation (in dark grey) and post-supplementation (in light grey). The range of post-supplementation minus pre-supplementation for erythrocyte, plasma and whole blood was −1.27 to 1.75, −0.53 to 1.13 and −0.07 to 2.56 for erythrocyte, plasma and whole blood, respectively.

**Figure 4:**
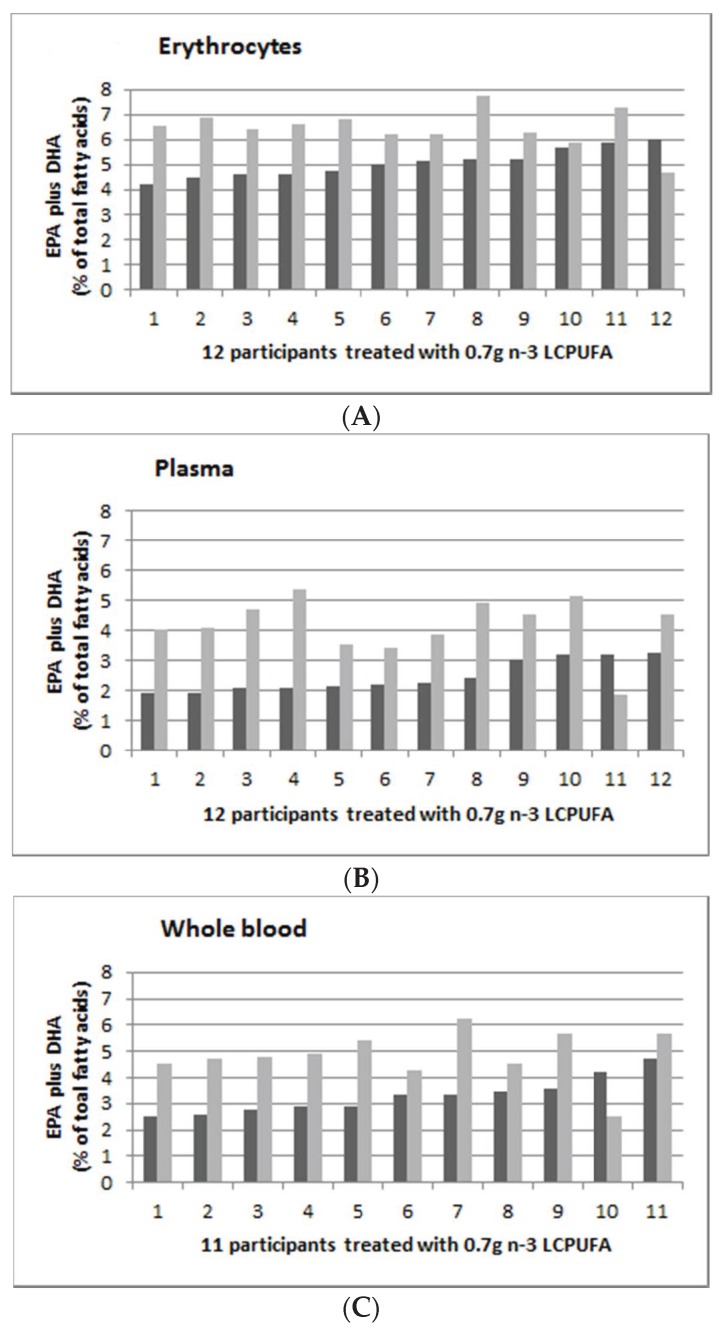
Omega-3 indices (EPA plus DHA expressed as percent of total fatty acids), in ascending order of 12 participants from the 0.7 g group (0.16 g EPA plus 0.54 g DHA per day). (**A**): Erythrocyte; (**B**): Plasma E; and (**C**): Whole blood. Dark grey is baseline (week 0) and light grey is post-supplementation (week 8).

Figure 4 shows the individual persons’ EPA and DHA (as % of total fatty acids) supplementation, with 0.7 g *n*-3 LCPUFA per day in erythrocytes (Figure 4A), plasma (Figure 4B) and whole blood (Figure 4C), with pre-supplementation (in dark grey) and post-supplementation (in light grey). The range of post-supplementation minus pre-supplementation for erythrocyte, plasma and whole blood was −1.3 to 2.52, −1.35 to 3.28 and −1.71 to 2.9 for erythrocyte, plasma and whole blood, respectively.

**Figure 5:**
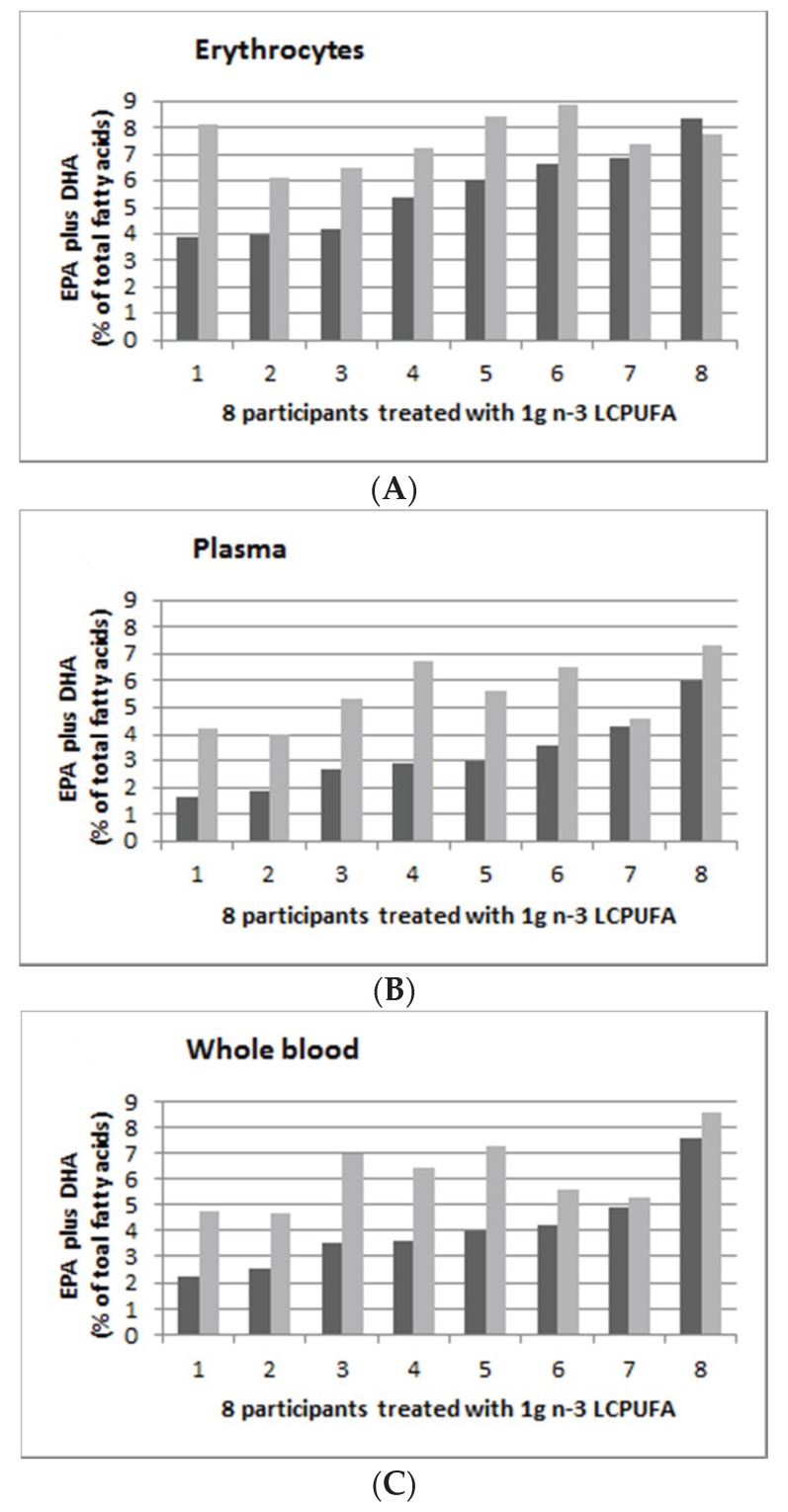
Omega-3 indices (EPA plus DHA expressed as percent of total fatty acids), in ascending order of 8 participants from the 1.0 g group (0.19 g EPA plus 0.81 g DHA per day). (**A**): Erythrocyte; (**B**): Plasma; and (**C**): Whole blood. Dark grey is baseline (week 0) and light grey is post-supplementation (week 8).

Figure 5 shows the individual persons’ EPA and DHA (as % of total fatty acids) supplementation with 1.0 g *n*-3 LCPUFA per day in erythrocytes (Figure 5A), plasma (Figure 5B) and whole blood (Figure 5C) with pre-supplementation (in dark grey) and post-supplementation (in light grey). The range of post-supplementation minus pre-supplementation for erythrocyte, plasma and whole blood was −0.55 to 4.23, 0.27 to 3.85 and 0.34 to 3.46 for erythrocyte, plasma and whole blood, respectively.

Figure 3, Figure 4 and Figure 5 suggest that there were greater increases in *n*-3 LCPUFA in those individuals with lower pre-supplementation EPA plus DHA levels, compared to those with higher pre-supplementation EPA plus DHA levels. There was a negative correlation between baseline erythrocyte EPA plus DHA and the change in erythrocyte EPA plus DHA levels (r = −0.1, *p* = 0.017), but there were no correlations in plasma (r = −0.01, *p* = 0.54) and whole blood (r = 0.01, *p* = 0.27).

## 4. Discussion

This study is a secondary analysis of a dose response study on *n*-3 LCPUFA supplementation and plasma lipids which has already been published [21]. In this secondary analysis, we have shown high individual variability in response to *n*-3 LCPUFA supplementation.

All participants included in the analysis were compliant (capsule count) with the consumption of the allocated capsules and therefore the individual variability in response to supplementation is unlikely to be attributed to the lack of compliance.

It has previously been reported that there is considerable variability in the erythrocyte EPA+DHA levels in response to *n*-3 supplementation [18]. Others have also reported that this happens in plasma and platelets [25] and neutrophils [26]. Certainly, plasma EPA and DHA levels are influenced by recent dietary intakes of EPA and DHA, whereas it is widely believed that erythrocyte levels of EPA and DHA are indicative of longer term status and reflect body tissues levels [27,28]. Despite this belief, several studies have shown that erythrocyte fatty acid compositions can be altered in less than seven days, by *n*-6 PUFA [29] and *n*-3 LCPUFA [30], perhaps through the exchange of fatty acids from plasma to erythrocytes and platelets. Therefore, it is likely that variability in response to supplementation seen in one blood compartment would be seen in all (erythrocytes, plasma and whole blood).

Studies have shown that the biological variability was lower in erythrocytes than in plasma using kinetic studies [31,32]. Other studies reveal that the extent of response to the supplements range from 0.4% to 6% increases to no increase, and in some cases decreases in the levels of EPA+DHA [18,25]. Since these reports of variability have been reported from different laboratories and in different countries, it is possible to rule out methodological issues and ethnicity as likely causes. One possible explanation for the variability is that people with different BMIs respond differently to the same dose of *n*-3 PUFA, leading to the idea that doses of *n*-3 should be adjusted for weight or BMI [33]. While this is a plausible explanation, it would not account for decreases in erythrocyte EPA+DHA after *n*-3 supplementation. The most likely explanation for such decreases is that the subjects failed to consume their capsules. Another possibility for the variation in the increases on EPA+DHA in erythrocytes is that there are differences between people in the uptake of *n*-3 PUFA into erythrocytes. To our knowledge, the mechanisms involved in the uptake of PUFA into erythrocytes have not been firmly established. There is evidence for the passive exchange of phospholipids between plasma and erythrocytes which allows renewal of erythrocyte PC [34], as well as acylation of lysoPC for uptake of free fatty acids from plasma [35]. This latter pathway would likely involve up to two enzymes, including lipoprotein lipase action on plasma lipoproteins to produce free fatty acids and then acylation of lysoPC to form PC. It is entirely possible that the expression of any one or both of those enzymes could be regulated differently in different subjects, in other words, under genetic control through single-nucleotide polymorphisms (SNPs).

The novelty of our current study is that we compared erythrocytes, plasma and whole blood from the same individuals. The individual variability in this study is high and comparable to previous studies where similar coefficients of variation (0.21) have been reported [17].

There are non-dietary factors that are associated with blood *n*-3 LCPUFA levels [15], including gender, age, alcohol consumption, smoking and BMI. As the original study was designed for pre-menopausal women, we only had one gender in this study and therefore cannot comment on the effect of gender in this study. Age had no effect (results not shown) possibly due to the narrow age range (18–39 years) in this study. Alcohol consumption had no effect (results not shown) and in the original study, smoking status was not collected. In terms of BMI, five previous studies showed a negative association of BMI and EPA plus DHA levels, whilst six previous studies showed no association [15]. In this study, dietary intake of EPA and DHA explained approximately 50% of the variance in blood EPA plus DHA levels. In our model 2, when we added BMI, it increased the variance of EPA plus DHA to 55%–58%.

In this small study, it appeared that the lower the pre-supplementation EPA plus DHA levels, the greater the increase in EPA plus DHA levels, especially as measured in erythrocyte EPA plus DHA, for reasons that are not clear. Published data in this context are limited, but our results are consistent with at least one previous report, which showed that as individuals baseline *n*-3 LCPUFA increased, the treatment-associated changes decreased [36]. Apart from diet and baseline EPA and DHA levels, the variability in response could be due to differences in digestion of the supplements, possibly due to differences in pancreatic lipase activity and/or gastrointestinal transport of lipids [37,38]. Certainly, consuming *n*-3 LCPUFA supplements with sufficient amounts of fat in the meal has the greatest bioavailability effect (up to three times higher [39], as fat in the meal stimulates fat digestion and hence increases the bioavailability of *n*-3 LCPUFA) [39]. Another possibly for some of these data is that there are some individuals who are non-responders to *n*-3 LCPUFA supplementation. It is possible that non-responders have gene-related impaired mechanisms for the uptake of *n*-3 LCPUFA from plasma lipoproteins. This lack of response was reported previously by Köhler et al. [17,18] An alternate explanation is that some individuals do not take their capsules for the study period, however this should show up on capsule recounts.

These results presented here emphasise the need to measure baseline blood EPA and DHA levels and post-supplementation levels in any trials which attempt to test the effect of supplemental EPA plus DHA on biological/physiological outcomes. Therefore, in studies which aim to assess the effect of *n*-3 LCPUFA, it is important to consider the guidelines offered by the ISSFAL Official Statement Number 6: The importance of measuring blood omega-3 long chain polyunsaturated fatty acid levels in research [16].

These results also support using an individualised dose of EPA and DHA in clinical trials, such that the target levels are reached prior to evaluating the outcomes of the trial [18,40].

### Strengths and Limitations

A strength of this study is the novelty in terms of reporting the individual variability in response to various doses of *n*-3 LCPUFA supplementation in erythrocytes, plasma and whole blood in the same individuals. Another strength of the study was controlling blood collection according to menses, minimizing the effects of hormones on fatty acid metabolism.

The limitations of this study include a small sample size and relatively short duration of intervention, although others have shown that an eight week intervention is long enough to increase erythrocyte *n*-3 LCPUFA [41] and we have shown significant results. However, it is premature to assume that these results apply to a larger sample size in Australia or in other countries, or in different ethnic groups. Another limitation is that this study was conducted only in women of childbearing age.

## 5. Conclusions

There is high individual variability in response to *n*-3 LCPUFA supplementation in women of child-bearing age. Further studies in different populations examining whether variations in response to supplements are consistent for individuals are needed, to fully understand the individual responses to *n*-3 LCPUFA supplementation.

## Figures and Tables

**Table 1:** Subject characteristics (*n* = 45) (values mean and standard deviation (SD)).

	Whole Group (*n* = 45)		0 g/day (*n* = 11)		0.35 g/day (*n* = 14)		0.7 g/day (*n* = 12)		1 g/day (*n* = 8)		*p* Value
	Mean	SD	Mean	SD	Mean	SD	Mean	SD	Mean	SD	
Age (years)	26.5	6.4	26.8	6.8	26.8	6.7	24.7	5.3	28.5	7.4	0.63
SBP (mmHg)	114	9.1	113	7.0	116	8.8	116	12.0	109	7.0	0.35
DBP (mmHg)	71	8.4	70	7.2	72	8.5	72	11.2	71	5.3	0.91
BMI	25	5.1	23	3.1	25	5.3	25	6.2	25	5.4	0.68

Abbreviations: BMI: Body mass index; DBP: Diastolic blood pressure; SD: Standard deviation; SBP: Systolic blood pressure.

**Table 2:** Baseline dietary intakes of macronutrients and fatty acids, as measured by food frequency questionnaire (median (IQR), *n* = 45).

	Whole Group	Placebo Group	Doses of DHA			*p* Value
	(*n* = 45)	0 g/day (*n* = 11)	0.35 g/day (*n* = 14)	0.7 g/day (*n* = 12)	1 g/day (*n* = 8)	
	Median (IQR)	Median (IQR)	Median (IQR)	Median (IQR)	Median (IQR)	
Energy (kJ/day)	6165 (4979, 8021)	6412 (5173, 8029)	5862 (4568, 7618)	7109 (5245, 8969)	5187 (3774, 6731)	0.28
Protein (g/day)	79 (59, 96)	86 (64, 101)	72 (57, 95)	88 (58, 112)	64 (55, 87)	0.50
Carbohydrate (g/day)	163 (134, 201)	172 (156, 195)	146 (117, 200)	192 (141, 241)	149 (102, 176)	0.12
Total fat (g/day)	59 (44, 77)	61 (44, 81)	59 (45, 78)	67 (45, 77)	43 (31, 62)	0.43
SFA (g/day)	24 (16, 28)	24 (20, 28)	24 (18, 31)	24 (18, 32)	15 (12, 24)	0.38
MUFA (g/day)	22 (15, 27)	23 (14, 33)	21 (17, 28)	25 (15, 27)	16 (11, 23)	0.47
PUFA (g/day) ^#^	9.4 (7.4, 13.3)	7.5 (5.3, 10.5) *	13.3 (8.3, 18.4)	9.6 (7.6, 11.3)	8.8 (6.3, 13.4)	0.01
LA (g/day) ^#^	7.5 (6.3, 11.3)	6.4 (4.8, 8.3) *	11.2 (7.3, 15.8)	7.9 (6.2, 9.3)	6.91 (5.25, 11.1)	0.01
ALA (g/day) ^#^	0.86 (0.64, 1.44)	0.77 (0.59, 0.86)	1.00 (0.62, 1.73)	0.91 (0.69, 1.39)	0.84 (0.59, 1.43)	0.35
AA (g/day) ^#^	0.15 (0.12, 0.23)	0.15 (0.09, 0.20)	0.16 (0.13, 0.33)	0.15 (0.09, 0.21)	0.16 (0.11, 0.24)	0.08
EPA (mg/day) ^#^	120 (51, 178)	80 (27, 122)	123 (65, 195)	90 (55, 212)	153 (55, 207)	0.60
DPA (mg/day) ^#^	89 (54, 149)	87 (41, 113)	87 (58, 178)	93 (52, 150)	13 (56, 178)	0.18
DHA (mg/day) ^#^	117 (56, 242)	117 (21, 186)	110 (56, 249)	136 (77, 237)	145 (40, 392)	0.28
*n*-3 LCPUFA (mg/day) ^#^	338 (186, 546)	255 (96, 425)	368 (197, 600)	279 (203, 628)	445 (160, 700)	0.21

Abbreviations: AA: Arachidonic acid; ALA: Alpha-linolenic acid; EPA: Eicosapentaenoic acid; DPA: Docosapentaenoic acid; DHA: Docosahexaenoic acid; IQR: Interquartile range; LA: Linoleic acid; LCPUFA: Long chain polyunsaturated fatty acids; MUFA: Monounsaturated fatty acids; PUFA: Polyunsaturated fatty acids; SFA: Saturated fatty acids. ^#^ Data from PUFA questtionnaire; * Significantly different from placebo group (*p* < 0.05) using one-way ANOVA followed by post-hoc Tukey-Kramer HSD.

**Table 3:** Erythrocyte, plasma and whole blood *n*-3 LCPUFA (mol %), at baseline and after supplementation with 0-1000 mg/day EPA plus DHA from DHA-rich tuna oil (*n* = 45) (values are median (IQR).

	0 g/day (*n* = 11)		0.35 g/day (*n* = 14)		0.7 g/day (*n* = 12)		1 g/day (*n* = 8)	
	0 Weeks	8 Weeks	0 Weeks	8 Weeks	0 Weeks	8 Weeks	0 Weeks	8 Weeks
Erythrocyte								
EPA	0.48 (0.40, 0.58)	0.50 ^a^ (0.33, 0.71)	0.52 (0.44, 0.72)	0.54 ^a^ (0.43, 0.64)	0.63 (0.59, 0.70)	0.71 ^a^ (0.67, 0.81)	0.89 (0.40, 1.19)	1.14 ^b^ (0.76, 1.37)
DHA	4.38 (3.57, 5.54)	3.97 ^a^ (3.46, 4.95)	4.12 (3.48, 4.78)	4.94 ^a^ (4.48, 5.25)	4.47 (4.03, 4.82)	5.70 ^b^ (5.55, 6.24)	4.80 (3.63, 5.64)	6.27 ^b^ (5.93, 7.15)
EPA+DHA	4.82 (3.97, 5.99)	4.68 ^a^ (3.86, 5.57)	4.74 (3.85, 5.32)	5.44 ^a^ (4.98, 5.75)	5.10 (4.62, 5.60)	6.47 ^b^ (6.22, 6.87)	5.69 (4.00, 6.83)	7.59 ^c^ (6.70, 8.38)
Plasma								
EPA	0.54 (0.49, 0.76)	0.65 ^a^ (0.47, 0.85)	0.71 (0.56, 0.81)	0.71 ^a^ (0.57, 0.84)	0.73 (0.62, 0.86)	1.07 ^b^ (0.87, 1.33)	0.82 (0.57, 1.44)	1.64 ^c^ (1.10, 2.45)
DHA	1.92 (1.33, 2.15)	1.54 ^a^ (1.39, 1.91)	1.82 (1.47, 2.00)	2.32 ^a^ (1.98, 2.47)	1.61 (1.43, 2.12)	3.14 ^b^ (2.71, 3.41)	2.24 (1.52, 2.70)	3.76 ^c^ (3.18, 4.45)
EPA+DHA	2.59 (1.88, 2.70)	2.33 ^a^ (1.97, 2.76)	2.52 (2.14, 2.74)	3.06 ^a^ (2.79, 3.35)	2.24 (2.10, 3.15)	4.30 ^b^ (3.60, 4.88)	2.94 (2.07, 4.13)	5.48 ^c^ (4.34, 6.68)
Whole blood								
EPA	0.59 (0.48, 0.68)	0.63 ^a^ (0.47, 0.84)	0.66 (0.50, 0.77)	0.68 ^a^ (0.54, 0.89)	0.72 (0.60, 0.85)	0.90 ^a^ (0.74, 1.14)	0.88 (0.59, 1.36)	1.42 ^b^ (1.03, 2.17)
DHA	2.78 (2.3, 3.2)	2.37 ^a^ (2.07, 2.96)	2.56 (2.16, 2.87)	3.28 ^a,b^ (2.80, 3.41)	2.57 (2.13, 2.84)	4.05 ^b^ (3.61, 4.53)	3.05 (2.10, 3.40)	4.41 ^b,c^ (3.88, 5.56)
EPA+DHA	3.42 (2.80, 3.87)	3.04 ^a^ (2.71, 3.81)	3.17 (2.86, 3.50)	3.94 ^a,b^ (3.55, 4.17)	3.32 (2.79, 3.57)	4.79 ^b^ (4.52, 5.67)	3.81 (2.78, 4.73)	6.03 ^c^ (4.91, 7.21)

No significant differences between baseline levels across the 4 groups, except 1 g group at 0 weeks, is significantly higher than placebo at 0 weeks (*p* = 0.049). ^a,b,c^ Different letters denote significant differences between the groups post supplementation, all *p* < 0.0001. Abbreviations: EPA: Eicosapentaenoic acid; DHA: Docosahexaenoic acid. Whole blood contains erythrocytes, platelets and plasma.

**Table 4:** Correlations of dietary EPA plus DHA (mg/day) with blood levels of EPA plus DHA, at baseline and post-intervention.

	Dietary Intake of EPA Plus DHA (g/day) Baseline		Dietary Intake of EPA Plus DHA (g/day) Post-Intervention	
Model 1	R^2^ adjusted	*p* value	R^2^ adjusted	*p* value
EPA plus DHA in erythrocytes (% of total fatty acids)	0.25	0.0003	0.48	<0.0001
EPA plus DHA in plasma (% of total fatty acids)	0.21	0.0009	0.50	<0.0001
EPA plus DHA in whole blood (% of total fatty acids)	0.20	0.0016	0.48	<0.0001
Model 2 (includes baseline BMI)	R^2^ adjusted	*p* value	R^2^ adjusted	*p* value
EPA plus DHA in erythrocytes (% of total fatty acids)	0.28	0.0003	0.55	<0.0001
EPA plus DHA in plasma (% of total fatty acids)	0.32	<0.0001	0.58	<0.0001
EPA plus DHA in whole blood (% of total fatty acids)	0.26	0.0010	0.58	<0.0001

Dietary intake of EPA plus DHA post-intervention explains approximately 50% of the variance of EPA plus DHA in the blood (erythrocytes, plasma and whole blood), whereas it only explained up to 25% of the variance at baseline.

## Abbreviations

*n*-3 LCPUFA	Omega-3 long chain polyunsaturated fatty acids
EPA	eicosapentaenoic acid
DPA	docosapentaenoic acid
DHA	docosahexaenoic acid
LA	Linoleic acid
AA	Arachidonic acid
ALA	Alpha-linolenic acid
EDTA	ethylenediaminetetraacetic acid
FAME	fatty acid methyl esters
BMI	body mass index
SBP	systolic blood pressure
DBP	diastolic blood pressure
IQR	Interquartile range
L MUFA	Monounsaturated fatty acids
PUFA	Polyunsaturated fatty acids
SFA	Saturated fatty acids
